# Early functional and therapeutic effect of reversed tumour shoulder prosthesis reconstruction after proximal humerus tumour resection

**DOI:** 10.3389/fsurg.2022.987161

**Published:** 2022-09-23

**Authors:** Shang Wang, Yi Luo, Yitian Wang, Yuqi Zhang, Taojun Gong, Chongqi Tu, Yong Zhou

**Affiliations:** Department of Orthopedics, West China Hospital, Sichuan University, Chengdu, China

**Keywords:** reversed tumour shoulder prosthesis, function, proximal humerus tumour, deltoid ending point reconstruction, retrospective study

## Abstract

**Introduction:**

Reconstruction of proximal humeral tumours after resection is still controversial. And there are few articles describing oncology patients' postoperative function after reversed tumour shoulder prosthesis reconstruction. We investigated the functional results of patients who underwent reversed tumour shoulder prosthesis, including those who did not preserve the deltoid ending point.

**Patients and methods:**

We retrospectively evaluated 16 patients with proximal humerus tumours who had undergone reversed tumour shoulder prosthesis. All patients underwent type Malawer I proximal humeral resection surgery and standard reverse tumour shoulder arthroplasty with a modular reverse shoulder prosthesis. We sutured the severed end of the deltoid to the brachialis muscle using the artificial patch for patients who had their deltoid ending point resected. Patients are rehabilitated and followed up according to our instructions.

**Result:**

All patients were followed up for a mean of 27.4 months (13–59), and their mean age was 45.9 years (15–74). The mean length of the humeral resection was 11.6 cm (5–15). The mean shoulder mobility was 122° (82°–180°) in forward flexion; 39° (31°–45°) in posterior extension; 102° (65°–172°) in abduction; 43° (30°–60°) in external rotation; 83° (61°–90°) in internal rotation, and a mean MSTS score of 77.9% (63.3%–93.3%). The mean DASH score was 20.8 (2.5–35.8). The mean VAS score was 0.9. For patients who had their deltoid ending point resected, the mean length of the humeral resection was 14.0 cm; the mean shoulder mobility was 109° in forward flexion; 37.8° in posterior extension; 102.0° in abduction; 38.3° in external rotation; 86.3° in internal rotation, and the mean MSTS score was 78.8%; the mean DASH score was 21.6; the mean VAS score was 1.0.

**Conclusion:**

Patients who underwent reverse tumour shoulder arthroplasty can achieve good early postoperative function, survival rate and low complication rate. In addition, patients who had their deltoid ending point removed also obtained good function after particular reconstruction.

## Introduction

The proximal humerus is the third most common site for primary bone and soft tissue tumours (1). Currently, preservation or salvage of shoulder function after surgery is still the most critical part of the treatment process for these patients. For many patients, resection of large areas of soft tissue is necessary to get negative surgical margins. Traditionally, traditional reconstruction methods have included anatomic endoprostheses, osteoarticular allografts, and allograft-prosthetic composites. Although each method preserves a portion of the shoulder joint function, the overall postoperative shoulder function was unsatisfactory for the patients, especially those with rotator cuff injuries (2). Over the past two decades, more and more surgeons have started to use reverse total shoulder arthroplasty(rTSA) to improve the function of patients after oncologic reconstruction of the proximal humerus (3). The reverse shoulder arthroplasty medializes and distalizes the centre of rotation of the shoulder, allowing the shoulder joint to complete abduction, forward flexion and external rotation through the deltoid muscle without the rotator cuff (4).

Few articles describe oncology patients’ postoperative function after reverse tumour shoulder prosthesis reconstruction. Reverse allograft-prosthetic composite reconstruction has achieved good outcomes for malignant tumours of the proximal humerus, and very few studies have begun to use modular prostheses (5, 6). But the preservation of the deltoid ending point is necessary for each method. We collected postoperative functional data from patients who underwent modular reverse tumour shoulder arthroplasty at our institution in the last five years and found that all patients achieved good postoperative function. Moreover, some patients with long humeral osteotomy lengths and without preserved deltoid ending points also achieved good function by reconstructing the deltoid.

## Patients and methods

We conducted a retrospective study of all patients who underwent reverse tumour shoulder arthroplasty for benign and malignant tumours at West China Hospital between 2017 and 2022. There were 16 patients (seven males and nine females). All patients had imaging evidence of a lesion in the proximal humerus (4 metastatic carcinomas, 4 chondrosarcomas, 3 osteosarcomas, 2 Giant-cell tumours of bone, 1 malignant neurinoma, 2 benign tumours). All were evaluated preoperatively with Computed Tomography (CT) and Magnetic Resonance Imaging (MRI) for the extent of soft tissue and bone involvement.

All patients underwent type I proximal humeral resection surgery (surgical classification system described by Malawer in 1991) and standard reverse tumour shoulder arthroplasty with a reverse shoulder prosthesis (Chunli Co, Ltd., Tongzhou, Beijing, China). The prosthesis consisted of a non-anatomical design with a hemispherical glenoid component and a con-cave matching cup on a stem fixed to the humerus (7).

All patients required partial or total removal of the rotator cuff to achieve a negative margin. The senior surgeon at our institution (C. T.) performed all the surgeries. The surgical approach was the pectoralis major deltoid approach. The affected proximal humerus was resected according to the preoperative design using vernier callipers to determine the length of the osteotomy, including *en bloc* of the surrounding soft tissues and muscles to achieve a negative margin. The deltoid ending point was removed in four of these patients due to extensive tumour invasion. The axillary nerve was preserved intact in all patients. Mark the long head of the pectoralis major, latissimus dorsi, and biceps brachii with sutures. And then, we implant a reverse shoulder joint prosthesis. Finally, we repair muscles marked by previous sutures to ensure the stability of the joint prosthesis. For the four patients who had their deltoid ending point removed, we sutured the severed end of the deltoid to the brachialis muscle using the artificial patch to ensure abduction muscle strength and adjusted it to the appropriate muscle tone. Postoperative antibiotics such as cefuroxime were routinely used to prevent infection for 24–48 h. A 45°–75° abduction brace was used for four weeks after surgery. Active functional exercise of the hand, wrist and elbow and small passive movements of the shoulder joint was started the day after surgery. Patients with reconstructed deltoid need to start abduction activity 6 weeks after surgery and other patients from postoperative day 2–7 according to the instructions of the senior surgeon. The brace was removed one month after surgery.

**Figure 1:**
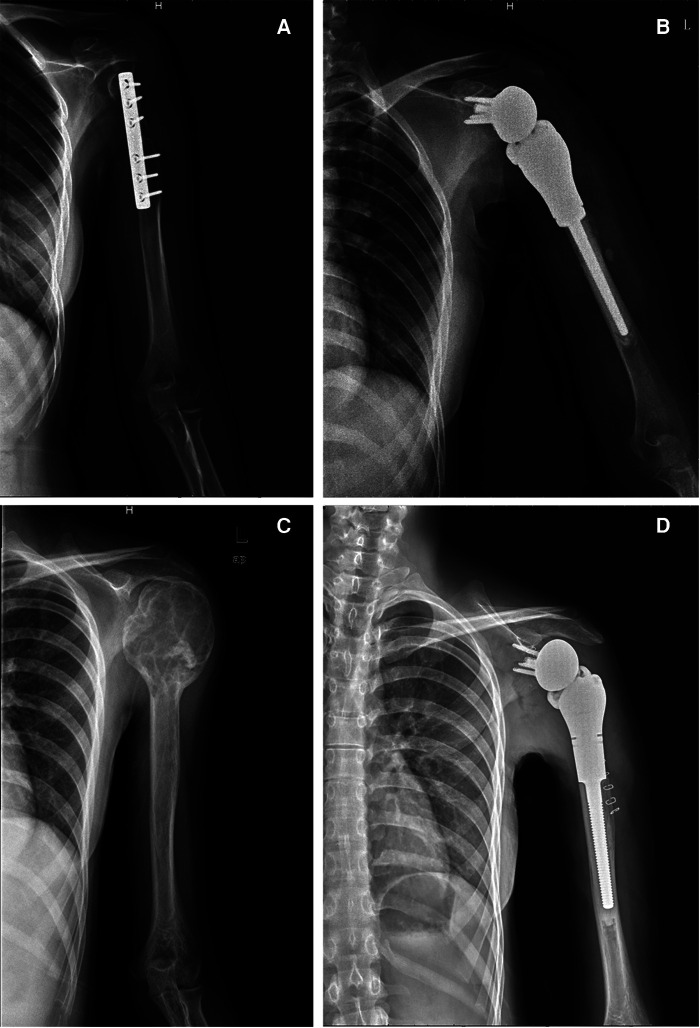
Imaging of two typical patients. (**A**) Preoperative x-ray of typical patient 1. (**B**) Postoperative x-ray of typical patient 1. (**C**) Preoperative x-ray of typical patient 2. (**D**) Postoperative x-ray of typical patient 2.

**Figure 2:**
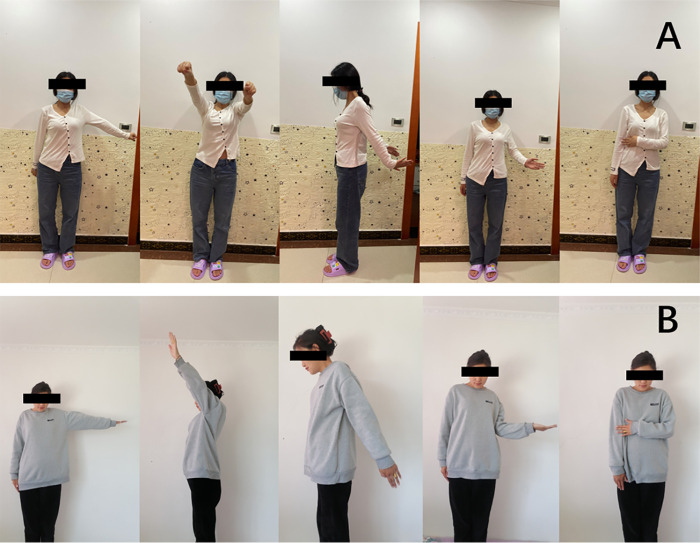
Postoperative functional photographs of two typical patients. (**A**) Postoperative functional photograph of typical patient 1. (**B**) Postoperative functional photograph of typical patient 2.

Patients were followed up at the second week, the first month, the third month, the sixth month, the first year, and every year after surgery. Follow-up included physical examination, x-rays, CT, shoulder mobility, and functional assessment of the shoulder. Shoulder function was assessed using Musculoskeletal Tumor Society(MSTS) scores (8) and Disabilities of the Arm, Shoulder and Hand (DASH) score (9). We used the Visual Analog Scale (VAS) score (10) for the pain to assess patients' pain. The first author measured shoulder mobility using a protractor on a photograph of the patient (Figures 1, 2). Additional information on the patient's age, gender, tumour pathology type, complications, length and extent of resection, and whether to remove the deltoid ending point were tallied according to the patient's inpatient medical record.

## Result

Two of the 16 patients with metastatic cancer (1 male, 1 female) died 14 and 21 months after surgery, and both patients were followed up to the time of death. All patients (7 male, 9 female) were followed up for a mean of 27.4 months (13–59), and their mean age was 45.9 years (15–74). The pathological type of the tumour is shown in Table 1. The length of the humeral resection ranged from 5–15 cm, with a mean of 11.6 cm.

**Table 1:** The data of all patients who underwent reverse tumour shoulder arthroplasty at West China Hospital between 2017 and 2022.

Patient	Age (yrs)	Sex	Follow-up (mths)	Pathological diagnosis	Humeral resection (cm)	Deltoid ending poingt	DASH score	MSTS score (%)	VAS score	Flexion (°)	Extension (°)	Abduction (°)	External rotation (°)	Internal rotation (°)
1	16	male	59	Chondroblastoma	12.0	preserve	19.1	80.0	1	161	45	122	43	81
2	66	female	52	Chondrosarcoma	9.0	preserve	23.3	76.7	0	169	39	155	51	90
3	55	male	45	Adenocarcinoma metastasis	13.0	preserve	2.5	90.0	0	161	38	93	60	90
4	15	female	41	Osteotelangiectasia	14.0	remove	17.5	73.3	0	98	32	91	33	83
5	26	female	33	Giant-cell tumor of bone	9.0	preserve	10	83.3	0	110	44	72	30	90
6	21	female	28	Giant-cell tumor of bone	10.0	preserve	16.6	93.3	0	180	45	164	60	90
7	20	male	24	Osteosarcoma	15.0	remove	15	93.3	0	171	44	172	48	88
8	65	male	22	Adenocarcinoma metastasis	13.5	preserve	35.8	63.3	0	91	31	92	32	61
9 (died)	74	male	21	Adenocarcinoma metastasis	14.0	preserve	24	72	2	100	38	94	35	80
10	65	female	20	Lipomyoma	5.0	preserve	25	63.3	2	91	44	73	48	90
11	36	female	19	Chondrosarcoma	7.0	preserve	16	86.7	1	141	40	121	54	90
12	50	male	17	Malignant neurinoma	12.0	preserve	26	70.0	1	86	32	66	32	70
13	58	female	15	Chondrosarcoma	14.0	remove	21.7	76.7	0	85	32	80	41	84
14	57	male	15	Chondrosarcoma	12.5	preserve	24	76.7	2	110	44	78	46	62
15 (died)	43	female	14	Kidney cancer metastasis	12.5	preserve	25	75	1	115	31	95	42	85
16	67	female	13	Osteosarcoma	13.0	remove	32	72.0	4	82	43	65	31	90
Mean	45.9		27.4		11.6		20.8	77.9	0.9	122	39	102	43	83
Mean of patients with deltoid ending point removed	40.0		23.3		14.0		21.6	78.8	1.0	109.0	37.8	102.0	38.3	86.3

DASH, Disabilities of the Arm, Shoulder and Hand; MSTS, Musculoskeletal Tumor Society; VAS, Visual Analog Scale.

The mean shoulder mobility at the last follow-up was 122° (82°–180°) in forward flexion; 39° (31°–45°) in posterior extension; 102° (65°–172°) in abduction; 43° (30°–60°) in external rotation; 83° (61°–90°) in internal rotation, and a mean MSTS score of 77.9% (63.3%–93.3%). The factor with the highest MSTS score was manual dexterity; the lowest was upper extremity weight-bearing capacity, which was significantly reduced in all patients.

The mean DASH score was 20.8 (2.5–35.8). The lowest scoring item was opening bottle caps, which all patients could do independently. The highest scoring item was lifting heavy objects over 5 kg, which is difficult for patients after shoulder arthroplasty.

The mean VAS score was 0.9. Eight patients did not have any pain and had a score of 0. The rest of the patients scored 1 or 2. Only one patient with a severed axillary nerve presented had pronounced pain. This patient's pain occurred mainly during activity; the VAS score was 4. The patient was also the most recent (13 months) to the last follow-up. The pain symptoms may resolve with a longer recovery time and rehabilitation training.

We counted the mean data from four patients with deltoid ending point removed. The mean length of the humeral resection was 14.0 cm. The mean shoulder mobility was 109° in forward flexion; 37.8° in posterior extension; 102.0° in abduction; 38.3° in external rotation; 86.3° in internal rotation, and the mean MSTS score was 78.8%; the mean DASH score was 21.6; the mean VAS score was 1.0.

As of the last follow-up, no patient had developed complications. Except for one patient with metastatic cancer with a lumbar metastasis, all patients had no distant metastasis of the tumour at follow-up. No patient had prosthesis loosening or periprosthetic fracture.

## Discussion

Tumours of the proximal humerus are the third most common bone tumour and the most common bone tumour of the upper extremity. Limb-preserving surgery for tumours of the proximal humeral has yielded good oncologic results (4, 11, 12). Since almost all tumour resections of the proximal humerus require resection of the rotator cuff insertions, although there are many types of shoulder reconstruction, the patient's postoperative upper extremity function, especially shoulder mobility, was poor after either surgery (Table 2) (13–18). Some investigators used reverse shoulder arthroplasty and allograft-prosthetic composite reverse shoulder arthroplasty to improve the function of the patients and achieved good results (Table 2) (5, 6, 19–21).

**Table 2:** Data from other studies on shoulder joint replacements.

Authors	Type of prosthesis	Patient (n)	MSTS score (%)	DASH score	Complication rate	Flexion (°)	Abduction (°)	External rotation (°)
Helmut et al	Endoprosthesis	15	70			20	20	20
Schmolders et al	Endoprosthesis	15	66.7			38	35	15
Raiss et al	Endoprosthesis	39	63.3			34	33	10
Potter et al	Endoprosthesis	16	69		44			
Matthew et al	Endoprosthesis	36	60		37	38		17
Zuo et al	Endoprosthesis	32			28	55.6	25	5
Marc El et al	APC	27	78			92		
Potter et al	APC	16	79		44			
Matthew et al	APC	17	69		63	55		21
Yao et al	Osteoarticular allograft	15				14.2	44	
Potter et al	Osteoarticular allograft	17	71		65			
Matthew et al	Reverse	20	67		15	76		27
Zuo et al	Reverse	20			15	95	110	25
Lazerges et al	Reverse	6	67	41	20	73	115	31
Nicolas et al	Reverse	10	67.5	29.5	30	122		
Matthew et al	Reverse APC	10	80		60	100		34

APC, allograft-prosthetic composite; MSTS, Musculoskeletal Tumor Society; DASH, Disabilities of the Arm, Shoulder and Hand.

Complications of shoulder replacement include shoulder instability, infection, nerve palsy, implant loosening, prosthesis dislocation, periprosthetic fracture, and for Allograft-Prosthetic Composite Reconstruction, nonunion and allograft fracture (4, 22–24). For rTSA, the most common complication is shoulder instability. The tendency to postoperative shoulder instability is also the biggest shortcoming of reverse tumour shoulder arthroplasty. Reconstructing as much muscle around the prosthesis as possible can somewhat prevent shoulder instability (4). Allograft-Prosthetic Composite Reconstruction can address the problem of easy loosening of the prosthesis after surgery because the composite prosthesis helps the bone grow. However, at the same time, the rate of postoperative complications is significantly higher because of the risk of nonunion, allograft fracture and bone resorption (16, 25). So, for Reverse Allograft-Prosthetic Composite Reconstruction, nonunion was the most common complication (24). The average rate of postoperative complications after traditional and reverse shoulder arthroplasty by other authors in Table 2 was 46.8% and 20%. As of the last follow-up, our patient had no significant complications. However, our mean follow-up time was too short, and a long follow-up observation is needed because some researchers have noted that the longer the postoperative period, the greater the probability of prosthetic loosening occurring (16).

Few articles describe reverse tumour shoulder arthroplasty's function after proximal humeral tumour resection. Furthermore, each article included a few patients. The rating scale used to assess patients' limb function was also inconsistent. Therefore, it is not easy to perform a large-scale statistical analysis. However, compiling and comparing the function of traditional surgical approaches (anatomic endoprostheses, osteoarticular allografts, and allograft-prosthetic composites) with that of reverse shoulder arthroplasty in different articles also revealed significant differences (1, 5, 13–21). For the traditional surgical approach and the reverse shoulder replacement, mean MSTS scores were 69.5% and 69.7%; mean complication rates were 46.8° and 28°; mean anterior flexion angles were 50.9° and 93.2°; mean abduction angles were 34.5° and 112.5°, and mean external rotation angles were 14.3° and 29.2°. The reverse shoulder arthroplasty was significantly superior to the conventional surgical approach regarding complications and joint mobility.

The 16 patients who underwent modular reverse tumour shoulder arthroplasty at our centre also had a good function. Except for two patients with metastatic cancer who died, all 14 patients could function adequately in daily life. All remaining patients did not experience severe pain, with eight patients experiencing no pain symptoms. The mean MSTS score of our patients is higher than the mean scores in other reverse shoulder replacement articles (Table 2). Our centre used the Disabilities of the Arm, Shoulder and Hand (DASH) score to assess patient function, and the score is lower than others' articles that also used the DASH score to assess the function of patients undergoing reverse shoulder arthroplasty at their institutions.

The conventional theory is that if the deltoid ending point cannot be preserved, the patient is not a candidate for reverse shoulder arthroplasty. We separately counted the postoperative function of patients who had their deltoid ending point removed. The results showed that these patients also achieved good postoperative function by reconstructing the adductor muscle by suturing the deltoid dissection to the brachialis muscle. This may suggest expanding the surgical indications for reversed tumour shoulder prosthesis. In addition, whether the deltoid stop is preserved or not, preservation of the deltoid muscle is critical for postoperative function in patients undergoing reverse tumour shoulder arthroplasty because patients with more deltoid muscle sacrifice generally had a poorer postoperative function. The study of Lazerges et al. included patients with more deltoid muscle sacrifice, and the mean DASH score was 41 (5, 21).

In patients with proximal humeral tumours, reverse tumour shoulder arthroplasty can achieve better postoperative function and lower postoperative complication rates than traditional shoulder arthroplasty, and both can achieve the same oncologic outcomes and patient satisfaction (26). Therefore, except for patients whose axillary nerve cannot be preserved and whose survival time is expected to be short, reverse tumour shoulder arthroplasty can be an excellent reconstructive modality after proximal humeral tumor resection. Moreover, patients with negative margins beyond the deltoid ending point may achieve good postoperative function with a particular deltoid ending point reconstruction.

Our study also has some limitations. The first is the short follow-up period. We should perform a long-term follow-up to obtain data on long-term postoperative function, complication rates, and other data. Secondly, the sample size was small and more patients undergoing reverse tumour shoulder arthroplasty should be included in the future to obtain more reliable data.

## Conclusion

The choice of reconstruction modality after tumor resection of the proximal humerus has been controversial. From the current follow-up results in our centre, patients who underwent reverse tumour shoulder arthroplasty can achieve good early postoperative function, survival rate and low complication rate. In addition, patients who had their deltoid ending point removed also obtained good function after particular reconstruction. Therefore, reverse tumour shoulder arthroplasty can be an option for shoulder reconstruction after resectioning the proximal humeral tumour. However, longer follow-up and larger sample sizes are also needed to obtain more reliable data.

## Data Availability

The original contributions presented in the study are included in the article/Supplementary Material, further inquiries can be directed to the corresponding author/s.
